# The complete mitochondrial genome of rubble crab *Daldorfia horrida* (Linnaeus, 1758) using next-generation sequencing

**DOI:** 10.1080/23802359.2019.1673255

**Published:** 2019-10-03

**Authors:** Mingqiu Yang, Yugui He, Hongtao Liu

**Affiliations:** Hainan Provincial Key Laboratory of Tropical Maricultural Technologies, Hainan Academy of Ocean and Fisheries Sciences, Haikou, China

**Keywords:** *Daldorfia horrida*, mitochondrial genome, phylogenetic analysis

## Abstract

In this study, the complete mitochondrial genome of the rubble carb *Daldorfia horrida* was determined using Illumina next-generation sequencing. The circular mitogenome is 15,737 bp, with 13 protein-coding genes (PCGs), 22 tRNA genes, two rRNA genes and one control region. The base composition is significantly biased (A, G, T, and C was 35.5%, 10.2%, 34.9%, and 19.4%, respectively) with A + T contents of 70.4%. Five microsatellites were identified in the mitogenome located in ND1, ND4, 16S genes and D-loop region. Phylogenetic tree showed that *D. horrida* is first clustered with *Pseudocarcinus gigas* and *Myomenippe fornasinii*, which revealed that Parthenopoidea has a close affinity with Eriphioidea in Heterotremata.

*Daldorfia horrida,* commonly known as horrid elbow crab or rubble crab, belongs to the family Parthenopidae (Tan et al. 1999). It is a large grotesque species, the convex surface of its body is extreme rugosity and sculpture, thickly covered with tubercles and eroded pits. It is widely distributed in the tropical and subtropical area of the Indian-Pacific Ocean, and inhabits from intertidal zone to the bottoms of rocks, sandy mud, or broken shells, and the shallow waters of coral reefs (Garth and Alcala 1977). Although it has been reported to be mildly toxic, but now still be consumed in some places in Hainan, China, and its toxicity needs to be further confirmed (Garth and Alcala 1977). Till now there are few studies on the species of Parthenopoidea. the mitochondrial genome of *D. horrida* as the first species which reports in Parthenopoidea will help to clarify its evolution and classification in the crustacean.

The samples were obtained from Qionghai, China (N19°18′48.37″, E110°40′20.21″), and stored in the marine crustacean specimen room (C20190606DH) in Hainan Academy of Ocean and Fisheries Sciences. The whole genome sequencing was conducted with 150 bp pair-end reads on the Illumine Hiseq Platform, and de novo assembled. The annotation was submitted to GenBank database (Accession Number: MN296513).

The complete mitogenome of *D. horrida* is 15,737 bp. The base content was 35.5% A, 10.2% G, 34.9% T, and 19.4% C. The 70.4% of (A + T) showed great preference to AT. It consists of 13 protein-coding genes (PCGs), 22 tRNA, two rRNA and one control region (D-loop). Four PCGs (ND1, ND4, ND4L and ND5), eight tRNA and two rRNA genes were located on the light strand, the others were encoded by the heavy strand.

22 tRNA genes in mitogenome of *D. horrida* vary from 62 bp to 72 bp. Three tRNA are present more than once: tRNA-Lue and tRNA-Asn have three copies respectively, and tRNA-Met has two copies. Functional replacement of reduced tRNA may be compensated by tRNA import or Superwobble (Rogalski et al. 2008; Salinas et al. 2008). The 12S rRNA is 698 bp, located between tRNA-Asn and tRNA-Ile; the 16S rRNA is 1315 bp, located between tRNA-Met and tRNA-Asn. Among 13 PCGs, only 8 PCGs use normal ATG or TAA as the start or stop codon respectively. The control region is 966 bp, located between tRNA-Gln and tRNA-Ile. Interestingly, we identified five microsatellites (SSR) in *D. horrida* mitogenome using MISA (Beier et al. 2017). Four types of SSRs are located in ND1, ND4, 16S rRNA and D-loop region.

A phylogenetic analysis was carried out based on mitogenome sequences of 35 Heterotremata species available in the GenBank to investigated the phylogenetic relationships of *D.horrida* using Maximum Likelihood (ML) method with 1000 bootstrap replicates. The result (Figure 1) showed that *D. horrida* is first clustered with *Pseudocarcinus gigas* and *Myomenippe fornasinii*, which revealed that Parthenopoidea is closer with Eriphioidea in Heterotremata. It is generally consistent with some previous studies (Lai et al. 2014; Basso et al. 2017; Tan et al. 2018).

**Figure 1.**
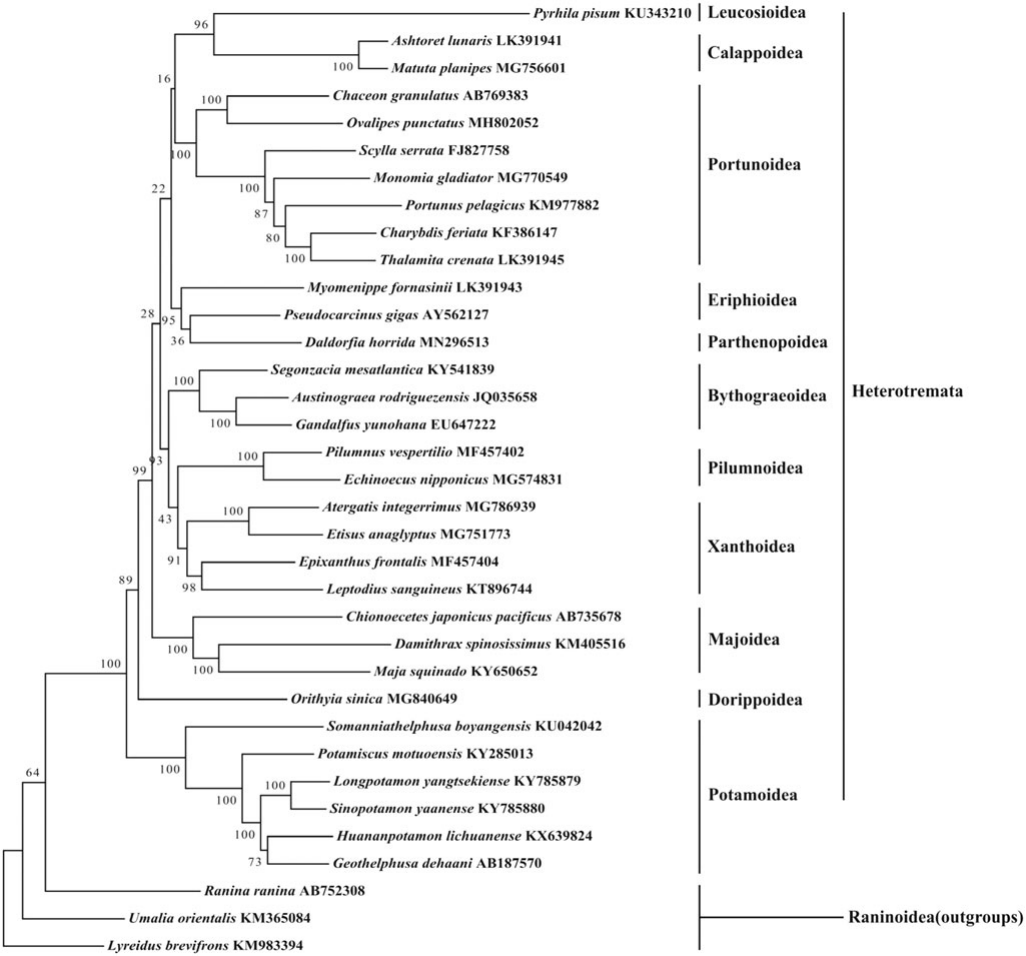
Phylogenetic tree of the complete mitogenome of 35 species in Heterotremata. *Lyreidus brevifrons, Umalia orientalis and Ranina ranina* were used as outgroups.
